# Dementia Care Management: Social Capital for Persons with Dementia that Live Alone

**DOI:** 10.1093/geroni/igab046.2427

**Published:** 2021-12-17

**Authors:** Adria Navarro, Vickie Avila, Lynne Conger

**Affiliations:** 1 Azusa Pacific University, Azusa, California, United States; 2 Alzheimer's Orange County, Irvine, California, United States

## Abstract

Social connections are critical for healthy aging (Ahn et al., 2020). Consistent findings in research for persons with dementia that live alone show that a natural support network is the gold-standard for success, as social capital enhances well-being. Therefore a community-based intervention was piloted as part of a national 'special populations grant' in partnership with the local Meals-on-Wheels program. Dementia care management provided by graduate social work interns sought to provide weekly supportive contacts. Quantitative data was collected at baseline and following the program through administration of standardized measurement tools for "thriving" and for "dementia quality of life." The sample to date (N=33) consists of a majority being white females (82.4%), with additional participants expected as the remaining enrolled complete the program. These early "completers" do not show significant changes in their specific "surviving to thriving" domains, yet they do report greater perceptions of quality in their life as a whole following the dementia care management intervention when comparing post data to their baseline (Chi Square 18.95, p=0.004). To date this intervention continues to be studied and suggests a positive impact is likely for older adults with dementia that live alone, as well as for the communities where they reside.

